# Heat Preconditioning of Nanofat Does Not Improve Its Vascularization Properties

**DOI:** 10.3390/cells14080581

**Published:** 2025-04-11

**Authors:** Francesca Bonomi, Ettore Limido, Andrea Weinzierl, Caroline Bickelmann, Emmanuel Ampofo, Yves Harder, Michael D. Menger, Matthias W. Laschke

**Affiliations:** 1 Institute for Clinical and Experimental Surgery, Saarland University, 66421 Homburg, Germany; francescabonomi.bonnie@gmail.com (F.B.); limidoettore@gmail.com (E.L.); andreaweinzierl@icloud.com (A.W.); caroline.bickelmann@uks.eu (C.B.); emmanuel.ampofo@uks.eu (E.A.); michael.menger@uks.eu (M.D.M.); 2 Department of Plastic Surgery and Hand Surgery, University Hospital Zurich, 8006 Zurich, Switzerland; 3 Department of Plastic, Reconstructive and Aesthetic Surgery and Hand Surgery, Centre Hospitalier Universitaire Vaudois (CHUV), 1005 Lausanne, Switzerland; yves.harder@chuv.ch; 4 Faculty of Biology and Medicine, University of Lausanne (UNIL), 1005 Lausanne, Switzerland

**Keywords:** heat preconditioning, nanofat, dermal substitutes, Integra^®^, survival, angiogenesis, vascularization, inflammation

## Abstract

Heat preconditioning has been shown to promote nutritive perfusion and tissue survival in autologous fat grafting as well as in flap and breast surgery. However, its impact on the vascularization properties of nanofat has not been investigated so far. Therefore, we exposed nanofat from donor mice to a temperature of 43 °C for 1 h and assessed the effects of this heat stress on cell viability and the expression of heat shock proteins (HSPs) and angiogenesis-related factors. Moreover, dermal substitutes seeded with heat-preconditioned and non-preconditioned control nanofat were implanted into dorsal skinfold chambers of recipient mice to study their vascularization and tissue integration in vivo by means of repeated intravital fluorescence microscopy, histology and immunohistochemistry. Heat preconditioning upregulated the expression of HSPs in nanofat without affecting cell viability. Moreover, it resulted in the downregulation of many pro-angiogenic factors and the increased expression of anti-angiogenic factors, indicating a shift towards an anti-angiogenic phenotype. Accordingly, implanted dermal substitutes seeded with heat-preconditioned nanofat exhibited a reduced vascularization and were not better integrated into the host tissue when compared to controls. These findings indicate that heat preconditioning cannot be recommended for enhancing the vascularization capacity of nanofat.

## 1. Introduction

Nanofat is a fluid fat derivative that can be rapidly generated by intra-operative mechanical emulsification and filtration of previously harvested autologous fat [1]. This approach destroys most adult adipocytes but preserves adipose-derived stem cells (ASCs), microvascular fragments and extracellular matrix components with a high content of growth factors and eventually regenerative capacity [1,2,3]. Therefore, nanofat has been reported to be effective in the treatment of scars, androgenic alopecia and skin wounds [4,5,6]. Moreover, it is currently applied in the field of facial rejuvenation [7].

Recently, we analyzed, in a preclinical setting, the regenerative effects of nanofat seeded on dermal substitutes [8]. The adequate vascularization of implanted dermal substitutes is important for their subsequent coverage with split-thickness skin grafts to reconstitute the physiological skin barrier and, thus, to protect patients from severe infections [9]. To achieve this, we seeded small samples of a clinically often used collagen-glycosaminoglycan dermal matrix with freshly generated nanofat from donor mice. After implantation of these nanofat-seeded samples into the dorsal skinfold chambers of the recipient animals, they finally exhibited improved vascularization and tissue integration when compared to non-seeded controls [8]. However, during the first 10 days after implantation, they still lacked ingrowing, functional, and blood-perfused microvessels. This raised the question whether the vascularization properties of nanofat can be further improved prior to its seeding onto dermal substitutes. For this purpose, preconditioning of nanofat may represent a promising approach that has not been investigated so far.

The preconditioning of tissues and cells is known as a strategy to enhance their resistance to subsequent stressors by first exposing them to different stimuli to induce protective cellular adaptations, such as the upregulation of stress-response genes and the release of cytokines [10,11,12]. Preconditioning has been widely tested for several clinical applications. In particular, heat preconditioning by short-term tissue exposure to supraphysiological temperatures has been shown to improve the outcome of flap and breast surgery as well as autologous fat grafting [13,14,15,16,17]. Stress by local heat induces the expression of heat shock proteins (HSPs), such as HSP-27, HSP-32, HSP-70 and HSP-90. In tumors, these HSPs are important drivers of angiogenesis [18]. Moreover, HSP-32, also called heme-oxygenase-1 (HO-1), is the main endogenous source of carbon monoxide that acts as a potent vasodilator enhancing nutritive tissue perfusion and cell viability [14,19,20]. Heat preconditioning also promotes the upregulation of vascular endothelial growth factor (VEGF) and, thus, stimulates the formation of new blood vessels [21].

Based on these observations, the aim of this study was to investigate the effects of heat preconditioning on cell viability as well as on the expression of HSPs and angiogenesis-related factors originating from nanofat. In addition, dermal substitutes seeded with heat-preconditioned nanofat were inserted in a mouse dorsal skinfold chamber to assess their vascularization and tissue integration and compared to dermal substitutes seeded with non-preconditioned control nanofat, as performed previously [8].

## 2. Materials and Methods

### 2.1. Animals

All animal experiments were performed in compliance with the National Institutes of Health (NIH) Guidelines on the Care and Use of Laboratory Animals (NIH publication #85-23 Rev. 1985) and the European legislation on the protection of animals (Directive 2010/63/EU). They were approved by the local authorities (permission number: 06-2022; State Office for Consumer Protection, Saarbrücken, Germany).

The inguinal fat pads were isolated from C57BL/6J wild-type mice for ex vivo analyses of nanofat. The animals exhibited a mean age of 4 months and an average body weight of 25 g. For in vivo experiments, the fat was harvested from green fluorescent protein (GFP)^+^ mice (C57BL/6-Tg (CAG-EGFP)131Osb/LeySopJ; The Jackson Laboratory, Bar Harbor, ME, USA). They exhibited a mean age of 4 months and an average body weight of 30 g. C57BL/6J wild-type mice with a mean age of 4 months and an average body weight of 25 g were equipped with dorsal skinfold chambers. They were housed one per cage to prevent mutual injuries due to the chambers at a temperature of 22–24 °C, a relative humidity of 50–60% and a 12 h light/dark cycle with free access to pellet chow (Altromin, Lage, Germany) and tap water.

### 2.2. Anesthesia

All procedures were performed under general anesthesia with carprofen for postoperative analgesia, as described previously in detail [8].

### 2.3. Generation and Heat Preconditioning of Nanofat

Nanofat was prepared, as previously described in detail [3], after sacrificing anesthetized donor mice by cervical dislocation. The nanofat from each donor mouse was equally divided into two samples. The first sample (heat-preconditioned nanofat; heat) was exposed to a constant temperature of 43 °C for 1 h in a heating incubator (Eppendorf ThermoMixer^®^ C, Eppendorf, Wesseling-Berzdorf, Germany). Thereafter, the preconditioned sample was allowed to recover for 3–6 h at room temperature before ex vivo analyses or used for in vivo experiments. The second sample (non-preconditioned nanofat; control) was not exposed to heat but was analyzed ex vivo after 3–6 h at room temperature or directly used for in vivo experiments, as outlined in detail in the following sections.

### 2.4. Ex Vivo Analysis of Nanofat

The viability of both control (*n* = 4) and heat-preconditioned nanofat (*n* = 4) was assessed by means of flow cytometry. The samples were dissociated into single-cell suspensions by Accutase^®^ (BioLegend, Fell, Germany) after 3 h at room temperature. Thereafter, the cells were washed in phosphate-buffered saline (PBS), resuspended in incubation buffer and stained for 15 min with propidium iodide (50 μg/mL; BD Biosciences, Heidelberg, Germany) and annexin V (100 μg/mL; ImmunoTools, Friesoythe, Germany). The cells were then analyzed in a flow cytometer (FACScan; BD Biosciences). The fraction of vital, apoptotic, necroptotic and necrotic cells was expressed as the percentage of all measured cells.

Moreover, after 6 h at room temperature, total RNA contained in control (*n* = 4) and heat-preconditioned nanofat samples (*n* = 4) was extracted using QIAzol lysis reagent (Qiagen, Hilden, Germany). The corresponding cDNA was synthesized from the total RNA by c-DNA Synthesis Kit (iScript cDNA Synthesis Kit; BioRad, Hercules, CA, USA), as described in the manufacturer’s instructions. We used SYBR-Green Supermix (SsoAdvanced Universal SYBR Green Supermix; BioRad) for quantitative real-time polymerase chain reaction (qRT-PCR), and the data analysis was carried out by a CFX96 RT-PCR System (BioRad). Murine β-actin was served as control. Forward and reverse primers (dissolved in RNase/DNase-free H_2_O) were used in a concentration of 500 nM. Primer sequences for qRT-PCR were coded as follows: 5′-AGAGTTCTGTCGCACCTATG-3′ (forward) and 5′-GGCTCAACTCTGGCTATCTC-3′ (reverse) for HSP-27; 5′-GCGGTACAAATCGGAAGATG-3′ (forward) and 5′-TTTGTCCTGCTCGCTAATCT-3′ (reverse) for HSP-70; 5′-ACCCTGACCATTGTGGATAC-3′ (forward) and 5′-CTCATCGTCGTTATGCTTCG-3′ (reverse) for HSP-90; 5′-AGGAGATAGAGCGCAACAAG-3′ (forward) and 5′-CTCGTGGAGACGCTTTACAT-3′ (reverse) for HSP-32/HO-1.

Additional samples of control (*n* = 4) and heat-preconditioned nanofat (*n* = 4) were fixed after 6 h at room temperature in 4% formalin, embedded in paraffin and cut into 3 µm-thick sections for histological and immunohistochemical analyses. Sections were stained with hematoxylin-eosin (HE). Moreover, sections were stained with a rabbit-anti-HO-1 antibody (1:100; Enzo Life Science, Lörrach, Germany) and a rabbit-anti-cleaved caspase-3 antibody (Casp-3; 1:100; Cell Signaling, Leiden, The Netherland). A goat anti-rabbit-peroxidase-labeled antibody (1:200; dianova GmbH, Hamburg, Germany) and a biotinylated goat-anti-rabbit IgG antibody (ready-to-use; Abcam, Cambridge, UK) were used as secondary antibodies. Thereafter, the numbers of Casp-3^+^ and HO-1^+^ cells were quantitatively assessed (5 regions of interest (ROIs) on 1 section per sample) by means of a BX53 microscope with the imaging software CellSens Dimension (version 1.11; Olympus, Hamburg, Germany).

Finally, a proteome profiler mouse angiogenesis array (R&D Systems, Bio-Techne; Wiesbaden-Nordenstadt, Germany) was performed to analyze pooled nanofat samples (*n* = 4 per group) after 6 h at room temperature according to the manufacturer’s instructions.

### 2.5. Seeding of Dermal Substitutes with Nanofat

As previously described [8], small samples were cut out of an Integra^®^ dermal regeneration template single layer without silicone sheet (Integra Life Sciences, Gent, Belgium) using a 4 mm biopsy punch (Kai Europe GmbH, Solingen, Germany). The samples were then placed for 10 min into a tube (Eppendorf, Hamburg, Germany) filled with either non-preconditioned nanofat or with heat-preconditioned nanofat for proper seeding [8].

### 2.6. Dorsal Skinfold Chamber Model

The effects of control and heat-preconditioned nanofat on the vascularization and integration of dermal substitutes were investigated in a mouse dorsal skinfold chamber model, as described previously in detail [8]. For this purpose, an Integra^®^ sample seeded with non-preconditioned nanofat (control; *n* = 8) or with heat-preconditioned nanofat (heat; *n* = 8) was positioned in the center of the chamber observation window for in vivo analyses.

### 2.7. Intravital Fluorescence Microscopy

The vascularization of the implants was repeatedly analyzed using intravital fluorescence microscopy over 14 days, as described previously in detail [8].

The microscopic movies were analyzed with CapImage (version 8.10.1; Zeintl, Heidelberg, Germany). This analysis included the following parameters: total number of perfused ROIs (%), functional microvessel density (cm/cm^2^) as well as diameter (μm), centerline red blood cell (RBC) velocity (μm/s), shear rate (s^−1^) and volumetric blood flow (pL/s) of individual microvessels in 8 ROIs located within the center (*n* = 4) and the border zones (*n* = 4) of each implant. Moreover, microhemodynamic parameters (diameter, centerline RBC velocity, shear rate and volumetric blood flow) and leukocyte-endothelial cell interactions (adherent leukocytes (mm^−2^) and rolling leukocytes (min^−1^)) of postcapillary and collecting venules within the host tissue were assessed in 4 different ROIs in direct vicinity to the implants [8].

### 2.8. Histology and Immunohistochemistry of In Vivo Samples

After the in vivo experiments, tissue samples were carefully excised, fixed in 4% formalin, embedded in paraffin and cut into 3 µm thick sections. HE staining was performed according to standard procedures. Additional sections were stained with antibodies against CD31, lymphatic vessel endothelial hyaluronan receptor (LYVE)-1 and GFP as well as collagen (Col) I, Col III, CD68, CD3 and myeloperoxidase (MPO), as described previously in detail [8].

The stained sections (1 section per sample) were used to measure microvessel density (mm^−2^), lymph vessel density (mm^−2^) as well as CD31^+^/GFP^+^ microvessels (%) and LYVE-1^+^/GFP^+^ lymph vessels (%). In addition, the total Col I and Col III ratio (implant/skin), the numbers of CD68^+^ macrophages (mm^−2^), CD3^+^ lymphocytes (mm^−2^) and MPO^+^ neutrophilic granulocytes (mm^−2^) were assessed in 2 ROIs in the border zones and 2 ROIs in the center of each implant.

### 2.9. Statistical Analysis

All data sets were first tested for normal distribution and equal variance (GraphPad Prism 10.1.2; GraphPad Software, San Diego, CA, USA). Thereafter, differences between the two groups were assessed by the unpaired Student’s *t*-test (parametric data) or Mann–Whitney rank sum test (non-parametric data). The values were given as mean ± standard error of the mean (SEM). A *p*-value of <0.05 was considered to indicate significant differences.

## 3. Results

### 3.1. Ex Vivo Characterization of Control and Heat-Preconditioned Nanofat

**Figure 1:**
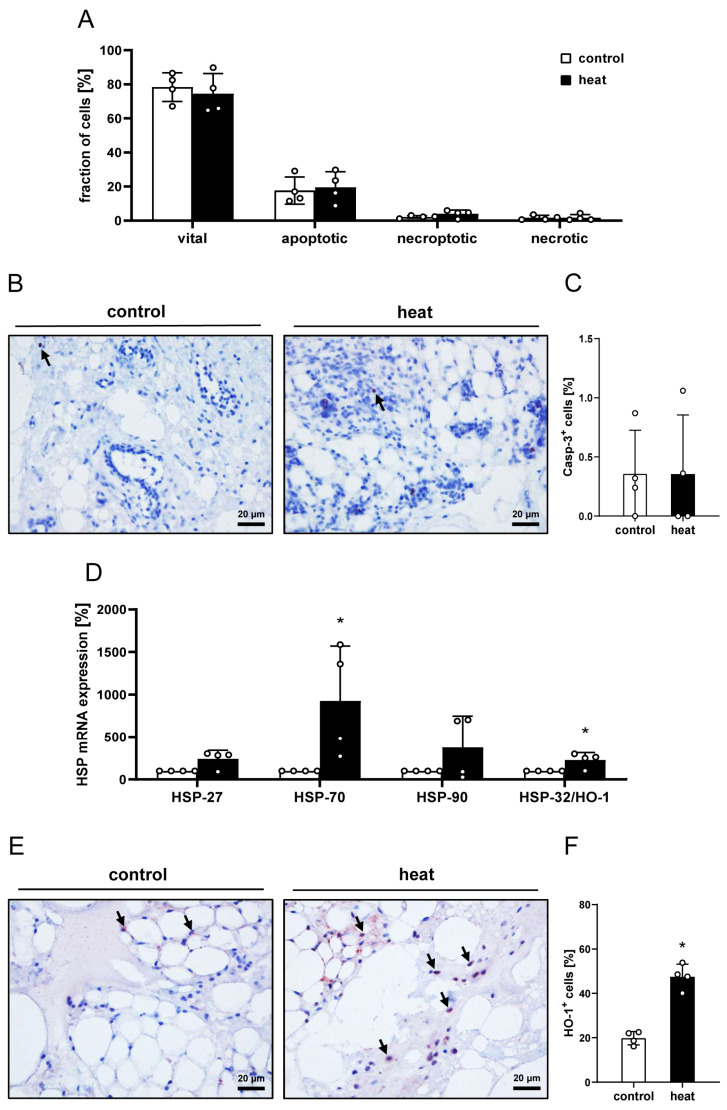
In vitro characterization of control and heat-preconditioned nanofat. (**A**) Fraction (%) of vital, apoptotic, necroptotic and necrotic cells in non-preconditioned nanofat (control; white bars, *n* = 4) and heat-preconditioned nanofat (heat; black bars, *n* = 4). Mean ± SEM. (**B**,**C**) Immunohistochemical assessment of Casp-3^+^ apoptotic cells ((**B**), arrows) and their quantitative analysis (**C**) in non-preconditioned nanofat (control; white bar, *n* = 4) and heat-preconditioned nanofat (heat; black bar, *n* = 4). Mean ± SEM. (**D**) mRNA expression (% of control) of HSP-27, HSP-70, HSP-90 and HSP-32/HO-1 in non-preconditioned nanofat (control; white bars, *n* = 4) and heat-preconditioned nanofat (heat; black bars, *n* = 4). Mean ± SEM; * *p* < 0.05 vs. control. (**E**,**F**) Immunohistochemical assessment of HO-1^+^ cells ((**E**), arrows) and their quantitative analysis (**F**) in non-preconditioned nanofat (control; white bar, *n* = 4) and heat-preconditioned nanofat (heat; black bar, *n* = 4). Mean ± SEM; * *p* < 0.05 vs. control.

In the first set of experiments, nanofat samples from donor mice were exposed ex vivo to a constant temperature of 43 °C for 1 h and compared to non-preconditioned nanofat, which served as control. Macroscopically, no differences between the two sample types were detected in terms of color or consistency. Moreover, flow cytometric analyses revealed that heat preconditioning of nanofat does not affect its viability (Figure 1A). In fact, heat-preconditioned and control nanofat exhibited comparable fractions of vital, apoptotic, necroptotic and necrotic cells (Figure 1A). This was confirmed by additional immunohistochemical staining of the apoptosis marker Casp-3, which did not show significant differences between sections of heat-preconditioned and control nanofat (Figure 1B,C).

In addition, qRT-PCR analyses revealed an upregulation of HSP gene expression as a response to heat stress to nanofat (Figure 1D). In line with this finding, immunohistochemically stained sections of heat-preconditioned nanofat exhibited a significantly higher number of HO-1^+^ cells when compared to controls (Figure 1E,F).

A proteome profiler mouse angiogenesis array was performed to analyze the expression of angiogenesis-related proteins in heat-preconditioned nanofat in comparison to non-preconditioned control nanofat. The array demonstrated that in heat-preconditioned nanofat the expression of many pro-angiogenic factors (22 out of 39) is downregulated, whereas the expression of most anti-angiogenic factors (10 out of 14) is upregulated when compared to control (Table 1). This indicates a shift in heat-preconditioned nanofat towards an anti-angiogenic phenotype.

### 3.2. In Vivo Microscopy of Nanofat-Seeded Implants

**Figure 2:**
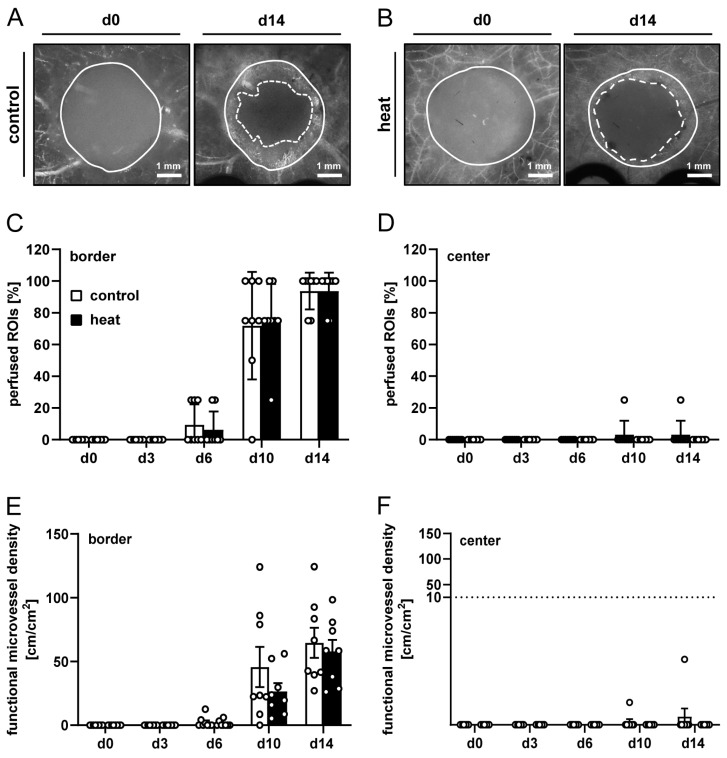
In vivo microscopy of nanofat-seeded implants. (**A**,**B**) Representative intravital fluorescence microscopic images of dermal substitutes seeded with non-preconditioned (control, (**A**)) and heat-preconditioned nanofat (heat, (**B**)) (implant border = closed line; border of non-vascularized implant area = broken line). (**C**–**F**) Perfused ROIs (%) (**C**,**D**) and functional microvessel density (cm/cm^2^) (**E**,**F**) in the border (**C**,**E**) and center (**D**,**F**) of dermal substitutes seeded with non-preconditioned (control; white bars, *n* = 8) and heat-preconditioned nanofat (heat; black bars, *n* = 8). Mean ± SEM.

In a second set of in vivo experiments, dermal substitutes seeded with heat-preconditioned or control nanofat were implanted into dorsal skinfold chambers to analyze their vascularization by means of repeated intravital fluorescence microscopy (Figure 2A,B). These analyses did not show marked differences between the two groups (Figure 2C–F). Six days after implantation, newly formed, blood-perfused microvascular networks could be detected in the border zones of the implants of both groups, which progressively increased in their density over time (Figure 2C,E). In the group of dermal substitutes seeded with heat-preconditioned nanofat, this functional microvessel density showed a tendency towards lower values on day 10 after implantation when compared to controls, albeit without reaching statistical significance (Figure 2E). Furthermore, almost no blood-perfused microvessels could be detected in the center of the implants in either group throughout the entire observation period of 14 days (Figure 2D,F). In line with these findings, individual microvessels within dermal substitutes seeded with heat-preconditioned or control nanofat did also not markedly differ in terms of diameter, centerline RBC velocity, shear rate and volumetric blood flow (Table 2).

**Figure 3:**
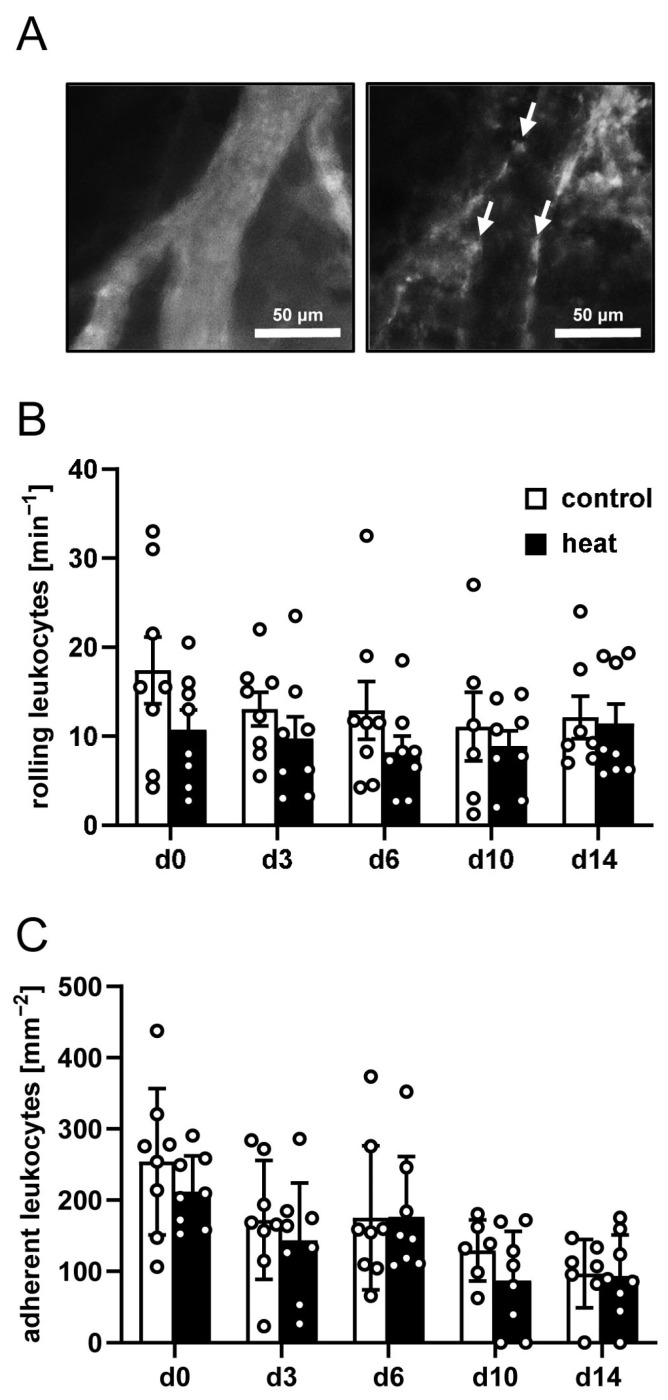
Interaction of leukocytes with the microvascular endothelium in response to nanofat-seeded implants. (**A**) Representative intravital fluorescence microscopic images of a venule next to an implanted dermal substitute seeded with non-preconditioned nanofat (blue light epi-illumination, contrast enhancement by 5% fluorescein isothiocyanate (FITC)-labeled dextran (left panel); green light epi-illumination, in situ staining of leukocytes with 0.1% rhodamine 6G (right panel); arrows = leukocytes). (**B**,**C**) Rolling leukocytes (min^−1^) (**B**) and adherent leukocytes (mm^−2^) (**C**) within venules next to dermal substitutes seeded with non-preconditioned (control; white bars, *n* = 8) and heat-preconditioned nanofat (heat; black bars, *n* = 8). Mean ± SEM.

To additionally investigate implant-induced inflammation, the interactions of leukocytes with the microvascular endothelium were quantified in venules next to the dermal substitutes seeded with heat-preconditioned or control nanofat. In both groups, these vessels presented with comparable microhemodynamic conditions (Table 3). Moreover, they did not show statistically significant differences in the numbers of rolling and adherent leukocytes during the in vivo experiments (Figure 3A–C).

### 3.3. Histological and Immunohistochemical Analysis of Nanofat-Seeded Implants

**Figure 4:**
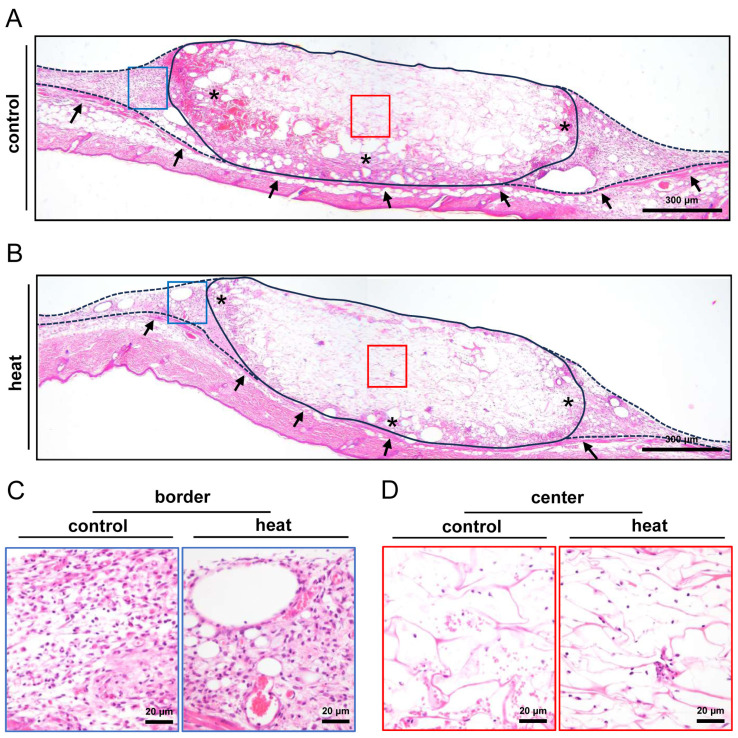
Tissue incorporation of nanofat-seeded implants. (**A**,**B**) HE-stained sections of implanted dermal substitutes seeded with non-preconditioned (control, (**A**)) and heat-preconditioned nanofat (heat, (**B**)) (implant border = closed line; border zone = broken line; ROIs in the border and center zones of the implants shown in higher magnification in (**C**,**D**) = blue and red frame; panniculus carnosus muscle = arrows; granulation tissue = asterisks). (**C**,**D**) Higher magnification of blue and red frames in (**A**) and (**B**) in the border (**C**) and center (**D**) zones of the implants.

**Figure 5:**
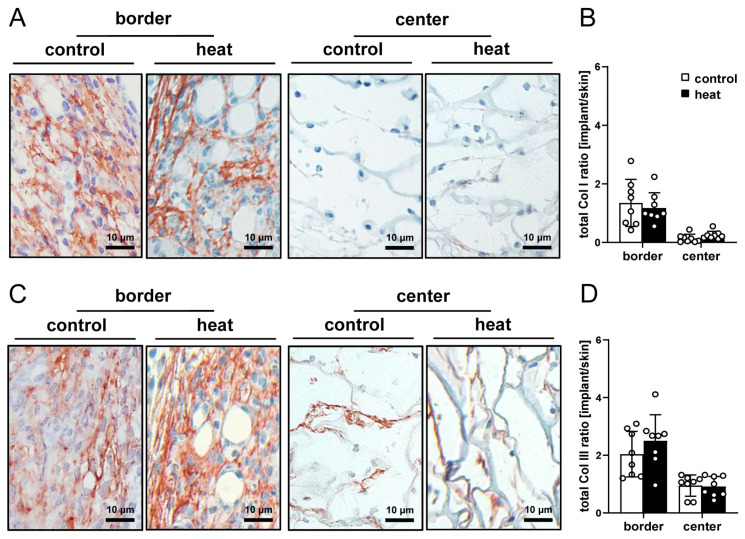
Collagen content of nanofat-seeded implants. (**A**,**C**) Immunohistochemical assessment of Col I (**A**) and III (**C**) in the border and center zones of dermal substitutes seeded with non-preconditioned (control) and heat-preconditioned nanofat (heat). (**B**,**D**) Total Col I (**B**) and Col III (**D**) ratio (implant/skin) in the border and center zones of dermal substitutes seeded with non-preconditioned (control; white bars, *n* = 8) and heat-preconditioned nanofat (heat; black bars, *n* = 8). Mean ± SEM.

After the in vivo experiments (day 14), the implants were investigated by means of histology and immunohistochemistry. The analysis of HE-stained sections showed a comparable tissue integration of dermal substitutes seeded with heat-preconditioned or control nanofat (Figure 4A–D). This was confirmed by the immunohistochemical assessment of the Col I and Col III content within the implants, which did not significantly differ between the two groups (Figure 5A–D).

**Figure 6:**
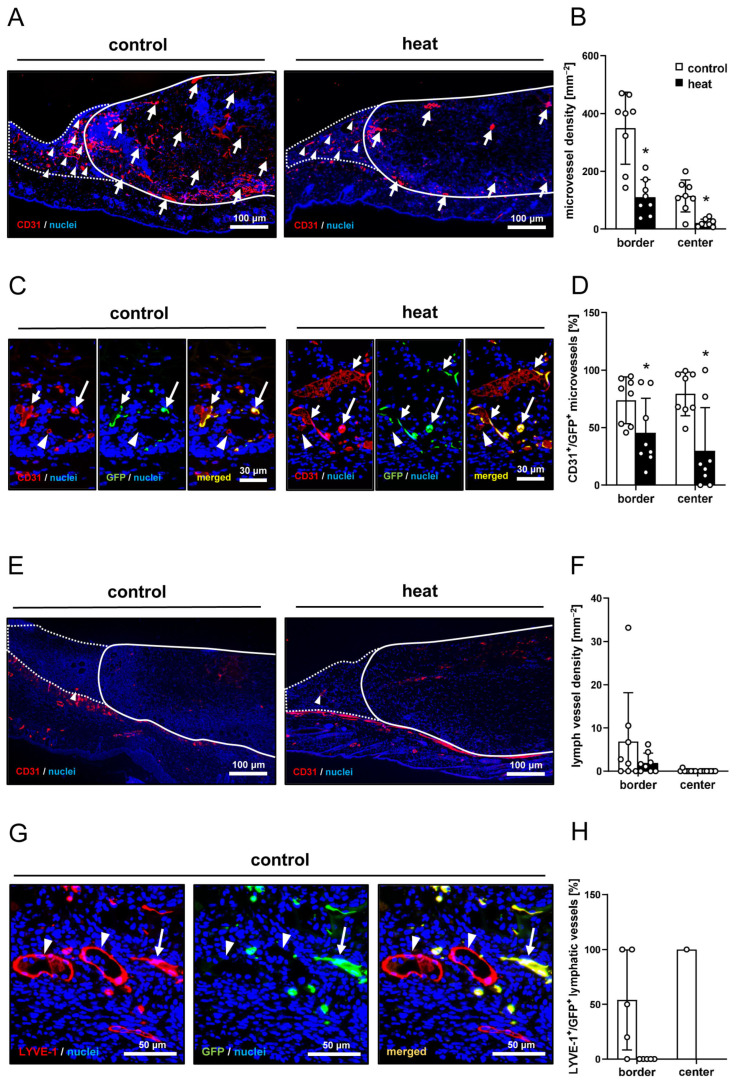
(**A**) Immunohistochemical assessment of CD31^+^ microvessels in the border zones (arrowheads) and the center (arrows) of dermal substitutes seeded with non-preconditioned (control) and heat-preconditioned nanofat (implant border = closed line; border zone = dotted line). (**B**) Microvessel density (mm^−2^) of dermal substitutes seeded with non-preconditioned (control; white bars, *n* = 8) and heat-preconditioned nanofat (heat; black bars, *n* = 8). Mean ± SEM. * *p* < 0.05 vs. control. (**C**) Immunohistochemical assessment of CD31^+^/GFP^−^ (arrowheads) and CD31^+^/GFP^+^ (arrows) microvessels within dermal substitutes seeded with non-preconditioned (control) and heat-preconditioned nanofat (heat). (**D**) CD31^+^/GFP^+^ microvessels (%) in the border and center zones of dermal substitutes seeded with non-preconditioned (control; white bars, *n* = 8) and heat-preconditioned nanofat (heat; black bars, *n* = 8). Mean ± SEM. * *p* < 0.05 vs. control. (**E**) Immunohistochemical assessment of LYVE-1^+^ lymph vessels in the border zones (arrowheads) of dermal substitutes seeded with non-preconditioned (control) and heat-preconditioned nanofat (heat) (implant border = closed line; border zone = dotted line). (**F**) Lymph vessel density (mm^−2^) of dermal substitutes seeded with non-preconditioned (control; white bars, *n* = 8) and heat-preconditioned nanofat (heat; black bars, *n* = 8). Mean ± SEM. (**G**) Immunohistochemical assessment of LYVE-1^+^/GFP^−^ (arrowheads) and LYVE-1^+^/GFP^+^ (arrows) lymph vessels in a dermal substitute seeded with non-preconditioned nanofat (control). (**H**) LYVE-1^+^/GFP^+^ microvessels (%) in the border and center zones of dermal substitutes seeded with non-preconditioned (control; white bars, *n* = 1–5) and heat-preconditioned nanofat (heat; black bars, *n* = 0–5). Mean ± SEM.

The immunohistochemical detection of CD31^+^ microvessels demonstrated that dermal substitutes seeded with heat-preconditioned nanofat exhibited a 3.5-fold lower microvessel density in both their border and center zones when compared to controls (Figure 6A,B). Of interest, GFP/CD31 co-stainings revealed that ~80% of the microvessels within implants seeded with control nanofat were GFP^+^, while in implants seeded with heat-preconditioned nanofat this fraction was significantly reduced (Figure 6C,D). In addition, immunohistochemical analyses of LYVE-1-stained sections showed that there were only a few lymphatic vessels surrounding the implants and almost none in the center with no significant differences in lymph vessel density between the two groups (Figure 6E,F). Most of these lymph vessels were GFP^+^ in the group of dermal substitutes seeded with control nanofat (Figure 6G,H). In contrast, none of the lymph vessels in the group of dermal substitutes seeded with heat-preconditioned nanofat exhibited a GFP^+^ signal (Figure 6H).

**Figure 7:**
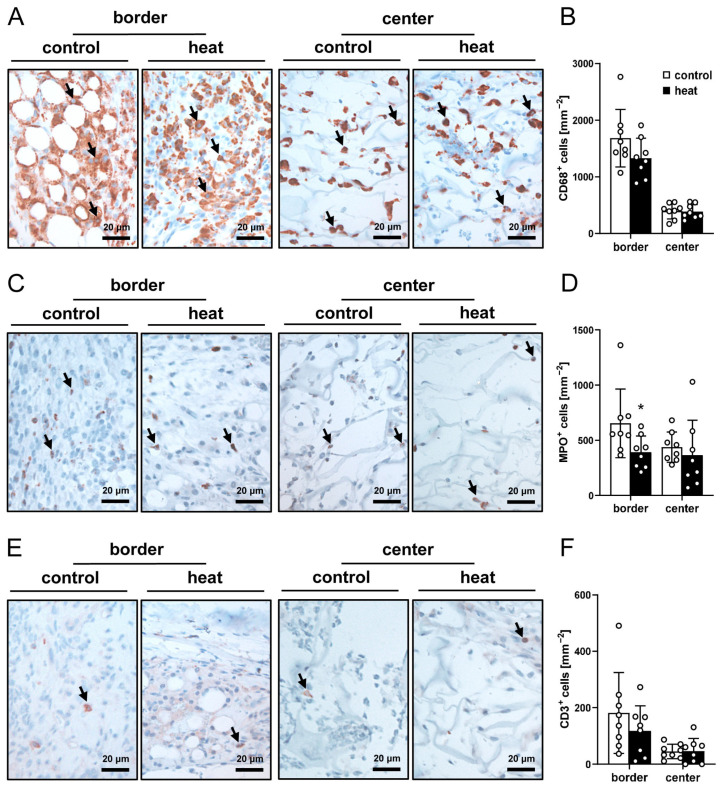
Immune cell infiltration of nanofat-seeded implants. (**A**,**C**,**E**) Immunohistochemical assessment of CD68^+^ macrophages ((**A**), arrows), MPO^+^ granulocytes ((**C**), arrows) and CD3^+^ lymphocytes ((**E**), arrows) in the border and center zones of dermal substitutes seeded with non-preconditioned (control) and heat-preconditioned nanofat (heat). (**B**,**D**,**F**) CD68^+^ macrophages (mm^−2^) (**B**), MPO^+^ granulocytes (mm^−2^) (**D**) and CD3^+^ lymphocytes (mm^−2^) (**F**) in the border and center zones of dermal substitutes seeded with non-preconditioned (control; white bars, *n* = 8) and heat-preconditioned nanofat (heat; black bars, *n* = 8). Mean ± SEM. * *p* < 0.05 vs. control.

Finally, CD68 (macrophages), MPO (neutrophilic granulocytes) and CD3 (lymphocytes) staining procedures were performed to quantify the immune cell infiltration of the implants. The quantitative analysis of these staining results did not show marked differences between the two groups (Figure 7A–F).

## 4. Discussion

Heat preconditioning has previously been shown to stimulate the upregulation of HSPs and angiogenic growth factors and, thus, to promote nutritive perfusion and survival of different tissues exposed to challenging hypoxic conditions [13,14,15,16,17,18,19,20,21]. In contrast to these findings, we demonstrated in the present study that heat preconditioning of nanofat does not further enhance its well-known regenerative properties but even reduces its in vivo vascularization capacity. Accordingly, we found that implanted dermal substitutes seeded with heat-preconditioned nanofat exhibit a significantly lower microvessel density and are equally integrated into the surrounding host tissue when compared to dermal substitutes seeded with non-preconditioned nanofat.

In recent decades, various heat preconditioning protocols have been proposed with different temperatures (between 40 and 44 °C or even 48 °C), exposure durations (20 s, 15–45 min or 1–2 h) and methods of heat supply (thermal chambers, blocks, pads, blankets or water baths) [13,14,17,22,23,24,25,26,27]. Of note, among the different temperatures 43 °C seems to be the most promising one [13,14,15,16,28,29,30], leading to an upregulated expression of HSPs and, thus, to stress protection already after 1 h of exposure [31,32,33,34]. Accordingly, we herein detected a significantly higher HSP-70 and HSP-32 gene expression and number of HO-1^+^ cells in nanofat that was exposed to 43 °C for 1 h in a heating incubator when compared to control. Furthermore, and of importance, it can be stated that this heating intervention did not affect the viability of the fat derivative. This indicates that the herein applied heat preconditioning approach is also suitable to effectively induce the previously described basic stress-responsive mechanisms in nanofat.

Several studies reported that the upregulation of HSPs, including HSP-70 and HSP-90, can promote blood vessel formation [17,35,36]. However, this was not the case in the present study. In fact, we found that heat-preconditioned nanofat even exhibits a reduced in vivo vascularization capacity when compared to non-preconditioned nanofat. This unexpected result may be explained by the mechanism of cellular quiescence, as it has been described to be induced by other stressors, such as serum deprivation [37,38]. Cellular quiescence is a reversible state, in which cellular activity and proliferation are prevented to improve stress resistance and survival [39,40,41]. When cells and tissues enter this state, survival mechanisms are often prioritized over other functions. In line with this concept, the overexpression of HSPs, such as HO-1/HSP-32, has been demonstrated to upregulate the cyclin-dependent kinase inhibitor p21, which increases resistance to apoptotic cell death, but decreases cell proliferation [42,43,44,45]. Moreover, we could show, by means of a proteome profiler mouse angiogenesis array, that the expression of many pro-angiogenic factors is downregulated in heat-preconditioned nanofat, whereas the expression of most anti-angiogenic factors is upregulated, indicating a shift towards an anti-angiogenic, quiescent phenotype. Accordingly, we also detected a markedly lower density of CD31^+^ microvessels in implanted dermal substitutes seeded with heat-preconditioned nanofat when compared to controls. This was associated with a significantly reduced fraction of GFP^+^/CD31^+^ microvessels within the border and center zones of the implants. The latter finding confirms a reduced angiogenic activity of the GFP^+^ microvascular fragments originating from the heat-preconditioned nanofat that had been seeded onto the dermal substitutes prior to their implantation into dorsal skinfold chambers of GFP^−^ recipient mice. Finally, it should be considered that heat preconditioning may not only induce cellular quiescence but may also negatively affect nanofat components that have been shown to contribute to blood vessel formation, such as extracellular matrix proteins [46]. For instance, from tumor studies it is well known that hyperthermia can promote the degradation of collagen [47]. However, since we did not detect marked differences in the Col I and Col III content of the implanted dermal substitutes, this may not primarily explain our finding of a reduced vascularization capacity of heat-preconditioned nanofat.

There is a close link between inflammation and angiogenesis. Immune cells have been shown to release various pro-angiogenic factors [48]. Furthermore, regenerative processes, such as wound healing, typically involve an initial inflammatory phase that is followed by a phase of blood vessel development [49]. To address this inflammation-driven angiogenesis in the present study, we also analyzed whether heat preconditioning changes the inflammatory activity of nanofat. For this purpose, we repeatedly assessed leukocyte–endothelial cell interactions in postcapillary and collecting venules in direct vicinity of nanofat-seeded dermal substitutes by means of intravital fluorescence microscopy and the immune cell infiltration of the implants at the end of the in vivo experiments by means of immunohistochemistry. However, no marked differences could be detected between dermal substitutes seeded with heat-preconditioned or non-preconditioned nanofat. Hence, it can be excluded that the reduced vascularization capacity of heat-preconditioned nanofat has been caused by an altered inflammatory activity.

Taken together, this is the first study that performed heat preconditioning of nanofat. The novel results demonstrate that exposure of nanofat to a temperature of 43 °C for 1 h does not enhance its vascularization capacity. However, one limitation of our study is the fact that we only tested one heat preconditioning protocol. Hence, it remains unclear whether another supraphysiological temperature or exposure duration would result in the same outcome. In addition, we primarily focused on the effects of heat preconditioning on angiogenic factors and blood vessels. However, the exposure to heat may also affect other biological components within nanofat, such as inflammatory cytokines, cellular lipids, extracellular matrix proteins or ASCs. Therefore, it may be interesting to clarify, in more detail, the fate of these components in response to heat. Moreover, our findings do not necessarily mean that other preconditioning approaches also fail in the case of nanofat. For instance, hypoxic preconditioning of fat grafts has already been shown to improve their vascularization and survival when compared to freshly generated controls [12,50,51]. This suggests that hypoxic preconditioning strategies should also be tested for nanofat in future studies.

## Figures and Tables

**Table 1:** Expression of pro- and anti-angiogenic proteins (% of control) in pooled heat-preconditioned nanofat. The data are presented in a descending order as mean of two technical replicates.

Protein	Expression (% of Control)
***Pro-angiogenic***	
HGF	564
KC/CXCL1/CINC-1/GRO-alpha	330
KGF/FGF-7	306
DLL4	299
ET-1	231
GM-CSF	211
PD-ECGF	207
AR	164
Proliferin	157
FGF acid/FGF-1/ECGF/HBGF-1	155
Coagulator Factor III/TF	147
EGF	125
VEGF/VPF	125
IGFBP-3	116
MMP-3	109
Ang-1	108
PIGF-2	104
MIP-1alpha	98
OPN	98
Leptin/OB	92
IL-10/CSIF	87
ANG	84
PDGF-AA	84
Cyr61/CCN1, IGFBP10	78
FGF basic/FGF-22	71
MMP-9	68
IL-1beta	55
Fractalkine/CX3CL1	53
IGFBP-1	52
IL-1alpha	45
VEGF B/VRF	45
SDF-1/CXCL12	43
MCP-1/CCL2	33
MMP-8	27
NOV/CCN3/IGFBP-9	25
CXCL 16	22
HB-EGF	21
Endoglin/CD105	19
IGFBP-2	6
***Anti-angiogenic***	
PTX3/TSG-14	637
ADAMTS1	204
Serpin F1/PEDF	204
DPP IV/CD26	195
Ang-3	166
PRL	160
IP-10/CXCL 10/CRG-2	151
PDFG-AB/BB	148
Endostatin/Collagen VIII	148
TIMP-1	111
CXCL4/PF4	69
Serpin E1/PAI-1	33
TIMP-4	30
TSP-2	16

ADAMTS: A Disintegrin And Metalloproteinase with Thrombospondin Motifs; ANG: Angiogenin; Ang: Angiopoietin; AR: Amphiregulin; CCL: Chemokine (C-C motif) Ligand; CCN: Cellular Communication network factor; CD: Cluster of Differentiation; CINC: Cytokine-Induced Neutrophil Chemoattractant; CRG: Cytokine-Responsive Gene; CSIF: Cytokine Synthesis Inhibitory Factor; CX3CL: Chemokine (C-X3-C motif) Ligand; CXCL: Chemokine (C-X-C motif) Ligand; Cyr: Cysteine-Rich Angiogenic Inducer; DLL: Delta-Like Ligand; DPP: Dipeptidyl Peptidase; ECGF: Endothelial Cell Growth Factor; EGF: Epidermal Growth Factor; ET: Endothelin; FGF: Fibroblast Growth Factor; GM-CSF: Granulocyte-Macrophage Colony-Stimulating Factor; GRO: Growth-Related On-cogene; HB-EGF: Heparin-Binding Epidermal Growth Factor; HBGF: Heparin-Binding Growth Factor; HGF: Hepatocyte Growth Factor; IGFBP: Insulin-Like Growth Factor Binding Protein; IL: Interleukin; IP: Interferon Gamma-Inducible Protein; KC: Keratinocyte Chemoattractant; KGF: Keratinocyte Growth Factor; MCP: Monocyte Chemoattractant Protein; MIP: Major Intrinsic Protein; MMP: Matrix Metalloproteinase; NOV: Nephroblastoma Overexpressed; OB: Obese; OPN: Osteopontin; PAI: Plasminogen Activator Inhibitor; PD-ECGF: Platelet-Derived Endothelial Cell Growth Factor; PDGF: Platelet-Derived Growth Factor; PEDF: Pigment Epithelium-Derived Factor; PF: Platelet Factor, PIGF: Placental Growth Factor; PRL: Prolactin; PTX3: Pentraxin-3; SDF: Stromal Cell-Derived Factor; TF: Tissue Factor; TIMP: Tissue Inhibitor of Metalloproteinases; TSG: Tumor Necrosis Factor-Induced Protein; TSP: Thrombospondin; VEGF: Vascular Endothelial Growth Factor; VPF: Vascular Permeability Factor; VRF: Vascular Remodeling Factor.

**Table 2:** Diameter (µm), centerline RBC velocity (µm/s), shear rate (s^−1^) and volumetric blood flow (pL/s) of microvessels within the border and center zones of dermal substitutes seeded with non-preconditioned nanofat (control; *n* = 8) and heat-preconditioned nanofat (heat; *n* = 8). Mean ± SEM; * *p* < 0.05 vs. control.; blanks indicate time points at which the implants lacked blood-perfused microvessels for microhemodynamic measurements.

	d0	d3	d6	d10	d14
***diameter (µm):***					
border: control	-	-	20.0 ± 4.5	19.4 ± 1.1	15.2 ± 0.9
heat	-	-	15.5 ± 2.4	17.2 ± 0.7	12.1 ± 0.8 *
center: control	-	-	-	-	20.9 ± 4.6
heat	-	-	-	-	-
***centerline RBC velocity (µm/s):***					
border: control	-	-	58.0 ± 17.4	96.1 ± 18.2	170.4 ± 14.9
heat	-	-	69.3 ± 11.3	134.7 ± 31.2	181.8 ± 28.8
center: control	-	-	-	-	131.8 ± 113.0
heat	-	-	-	-	-
***shear rate (s^−1^):***					
border: control	-	-	23.0 ± 4.2	43.5 ± 8.4	113.6 ± 19.2
heat	-	-	39.3 ± 13.2	69.3 ± 15.2	126.4 ± 31.9
center: control	-	-	-	-	63.8 ± 57.5
heat	-	-	-	-	-
***volumetric blood flow (pL/s):***					
border: control	-	-	15.7 ± 10.7	20.1 ± 5.0	20.4 ± 3.1
heat	-	-	7.8 ± 1.3	30.6 ± 10.7	15.4 ± 2.5
center: control	-	-	-	-	21.7 ± 15.5
heat	-	-	-	-	-

**Table 3:** Diameter (µm), centerline RBC velocity (µm/s), shear rate (s^−1^) and volumetric blood flow (pL/s) of postcapillary and collecting venules in direct vicinity to dermal substitutes seeded with non-preconditioned nanofat (control; *n* = 8) and heat-preconditioned nanofat (heat; *n* = 8). Mean ± SEM; * *p* < 0.05 vs. control.

	d0	d3	d6	d10	d14
***diameter (µm):***					
control	42.5 ± 2.3	38.7 ± 1.4	37.8 ± 1.6	35.1 ± 1.4	33.0 ± 2.0
heat	36.6 ± 1.4	34.4 ± 1.3	37.0 ± 1.3	36.2 ± 1.3	38.9 ± 1.3 *
***centerline RBC velocity (µm/s):***					
control	516.0 ± 61.1	522.9 ± 71.7	608.2 ± 75.3	512.8 ± 74.2	440.5 ± 125.0
heat	651.0 ± 97.6	674.0 ± 42.7	667.6 ± 86.1	658.1 ± 103.3	714.9 ± 109.2
***shear rate (s^−1^):***					
control	97.3 ± 8.7	98.3 ± 10.6	134.9 ± 20.1	112.5 ± 13.4	101.4 ± 28.0
heat	144.0 ± 22.4	157.3 ± 11.2 *	146.2 ± 20.2	148.7 ± 22.4	152.1 ± 25.0
***volumetric blood flow (pL/s):***					
control	510.9 ± 110.6	450.8 ± 86.7	438.5 ± 66.9	347.2 ± 69.1	292.5 ± 86.3
heat	445.2 ± 72.4	423.8 ± 42.2	481.8 ± 68.8	436.7 ± 79.4	553.8 ± 76.8 *

## Data Availability

The data are freely available upon request.

## Abbreviations

The following abbreviations are used in this manuscript:

ASC	Adipose-derived stem cell
Casp-3^+^	Caspase-3-positive
Col	Collagen
FITC	Fluorescein isothiocyanate
GFP	Green fluorescent protein
HE	Hematoxylin-eosin
HO-1	Heme-oxygenase-1
HSP	Heat shock protein
LYVE	Vessel endothelial hyaluronan receptor
MPO	Myeloperoxidase
NIH	National Institutes of Health
PBS	Phosphate-buffered saline
qRT-PCR	Quantitative real-time polymerase chain reaction
RBC	Red blood cell
ROI	Region of interest
VEGF	Vascular endothelial growth factor
